# A High-Generalizability Machine Learning Framework for Analyzing the Homogenized Properties of Short Fiber-Reinforced Polymer Composites

**DOI:** 10.3390/polym15193962

**Published:** 2023-09-30

**Authors:** Yunmei Zhao, Zhenyue Chen, Xiaobin Jian

**Affiliations:** 1 School of Aerospace Engineering and Applied Mechanics, Tongji University, Shanghai 200092, China; 2230883@tongji.edu.cn; 2 Department of Aeronautics and Astronautics, Fudan University, Shanghai 200433, China

**Keywords:** short fiber-reinforced polymer composite, ensemble machine learning, homogenized properties, SHAP interpretation, model generalizability

## Abstract

This study aims to develop a high-generalizability machine learning framework for predicting the homogenized mechanical properties of short fiber-reinforced polymer composites. The ensemble machine learning model (EML) employs a stacking algorithm using three base models of Extra Trees (ET), eXtreme Gradient Boosting machine (XGBoost), and Light Gradient Boosting machine (LGBM). A micromechanical model of a two-step homogenization algorithm is adopted and verified as an effective approach to composite modeling with randomly distributed fibers, which is integrated with finite element simulations for providing a high-quality ground-truth dataset. The model performance is thoroughly assessed for its accuracy, efficiency, interpretability, and generalizability. The results suggest that: (1) the EML model outperforms the base members on prediction accuracy, achieving $R^{2}$ values of 0.988 and 0.952 on the train and test datasets, respectively; (2) the SHapley Additive exPlanations (SHAP) analysis identifies the Young’s modulus of matrix, fiber, and fiber content as the top three factors influencing the homogenized properties, whereas the anisotropy is predominantly determined by the fiber orientations; (3) the EML model showcases good generalization capability on experimental data, and it has been shown to be more effective than high-fidelity computational models by significantly lowering computational costs while maintaining high accuracy.

## 1. Introduction

Applications of short fiber-reinforced polymer composites (SFRPCs) have been rapidly growing in fields such as bionic manufacturing, automotive, and aviation industries due to their lightweight, high strength, and ease of processing and manufacturing [1,2]. Significant advantages of SFRPCs over continuous fiber composites are that they are easily produced materials, which are more suitable for rapid and high-volume manufacturing in complex geometries with lower expenses.

The mechanical performance of SFRPCs strongly depends on the microstructural parameters, such as the Young’s modulus and Poisson’s ratio of the fibers and matrix, fiber dimensions and volume content, and the alignment distribution of fibers [3]. In particular, the orientation of fibers and the number of fibers aligned with the loading conditions can be customized by the injection molding process in manufacturing [1]. Engineers and scientists can evaluate materials’ functional characteristics before manufacturing, cut down on pointless experimental testing, streamline the entire design process, and lay a crucial foundation for expanding their applications and improving material performance by accurately predicting the mechanical performance of SFRPCs.

The orientation distribution of the fibers of SFRPCs can be experimentally examined using microscopic techniques like optical diffraction, electron microscopy, and X-ray radiography [4,5]. Image-informed statistical models are accordingly proposed to address the fiber distributions such as the diffusion orientation distribution function [6]. However, the complexity and diversity of fiber orientation states also present the primary challenge in aspects of constitutive relations [7], computational models [8], and numerical implementations [9].

Great efforts have been devoted to performing multiscale simulations utilizing Representative Volume Element (RVE) models to involve more accurate distributed fibers [10]. RVEs often fail to effectively reflect the intricate spatial fiber orientation, and increasing algorithm difficulties occur with rising fiber volume content [11,12]. The high computational time cost of Finite Element Analysis (FEA) presents another significant hurdle in simulations. The two-step homogenization method is proposed as an alternative effective approach [3,13]. The hybrid framework employs an Orientation Averaging (OA) approach to derive the mechanical properties on the basis of analysis of RVEs involving unidirectional fibers. This method significantly reduces modeling complexity and offers broader applicability. Nevertheless, it still cannot avoid the time-consuming nature of finite element calculations [14].

In recent years, machine learning (ML) techniques have been widely adopted in relevant fields of biochemistry [15], material science [16,17], and mechanical performance analysis [18,19]. with an end-to-end prediction paradigm, using a simulated dataset is commonly reported [19,20]. As suggested in [21], by providing enormous amounts of relevant data, numerical modeling naturally complements the ML technique and aids in creating reliable data-driven models. Simulation data with composite RVEs have been widely used to develop ML surrogates for fast prediction of the homogenized properties of composites [22,23,24].

The model selection is the key to determining the model’s performance, which varies depending on the complexity and heterogeneity of the microstructures. The simplest tree models and supported vector regressions (SVR) are efficient in dealing with homogenized elastic properties of 2D materials with idealized well-arranged microstructures [25]. Meanwhile, deep neural networks (DNN) have demonstrated efficiency in discovering distinct structures and learning the nonlinear relationship between the feature vector of microstructures and the expected effective mechanical properties [14,26,27]. Advanced image-based convolutional neural networks (CNN) and generative adversarial networks (GAN) display their strong capability in predicting the stress–strain curve of composites directly from RVEs that embed cracks and voids [28,29].

For composites with short fiber inclusions, CNN and DNN models are constantly used to investigate homogenized properties from the three-dimensional diverse spatial microstructures of fibers. Ref. [30] suggested a CNN model that takes the 2D RVE image as input with a different range of Young’s modulus of carbon fibers and neat epoxy, and obtains output as a visualization of the stress components. Ref. [14] utilized a DNN model that incorporates a micromechanical model to investigate the homogenized elastic properties of short fiber-reinforced composite. On one hand, training neural networks, particularly the CNN algorithm, is very time-consuming. On the other hand, existing works pay more attention to finding super-machine-learning models with capabilities beyond human limits while ignoring the key interpretability [31,32], resulting in the unknown mechanism of how the features modulate the machine-learning models. Lack of interpretability makes the data-driven models untrustworthy and would be problematic in model generalization [33,34].

The selection of appropriate machine learning models and efficient explanation strategies are receiving more attention [35]. Ensemble machine learning (EML) [36] is a general meta-approach machine learning that seeks better predictive performance by combining the predictions from multiple weak simple learners, such as tree models, SVR, and Gradient Boosting models. The EML model has demonstrated its application in accelerating composite material performance investigation [37,38] and optimization design [39,40]. Regarding the model interpretability methods, the SHapley Additive exPlanations (SHAP) method has gained increasing popularity, which explains how each feature influences the model and allows local and global analysis [41]. SHAP analysis has offered valuable insights into the disciplinary fields of composite performance analysis [19,37,42,43] and sensor fault detection [44], as well as manufacturing processes [45].

In this study, we propose an interpretable and high-generalizability data-driven model for accurately predicting the homogenized mechanical properties of SFRPCs. We utilize a staking algorithm of three base learners of ET, XGBoost, and LGBM. The dataset is constructed by numerical simulations using composite RVEs that are integrated with the two-step homogenization method. We comprehensively assess the model’s accuracy, efficiency, interpretability, and generalization capability. In particular, a SHAP analysis is performed to extensively evaluate the influence features and the underlying mechanisms on predictions. The model’s capacity to generalize is thoroughly investigated by including experimental data that take into account the real fiber distribution of SFRPCs as reported in the literature [46,47].

## 2. Material and Method

### 2.1. Two-Step Homogenization Procedure

#### 2.1.1. Orientation Tensor for Describing Fiber Distribution

**Figure 1:**
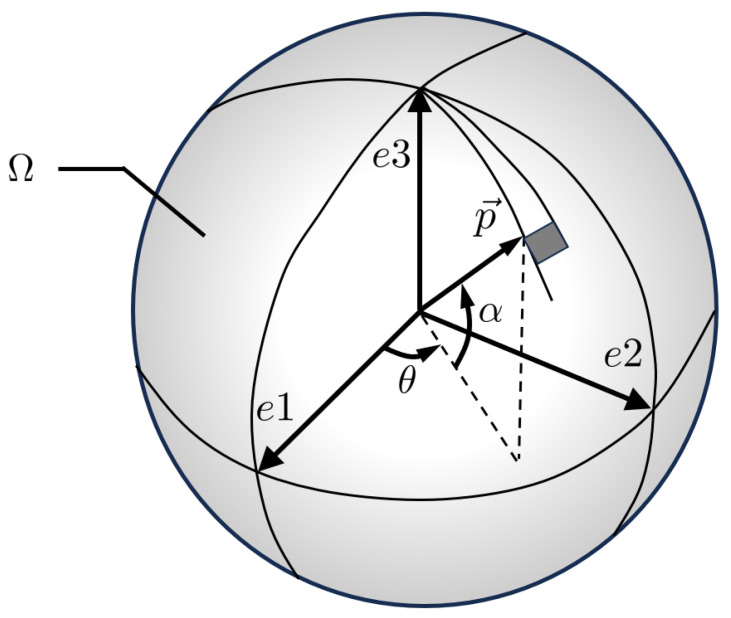
The probability distribution of fibers in a unit sphere Ω the one fiber direction can be expressed using two angle values of α and θ.

Figure 1 depicts the fiber orientation within a unit sphere Ω. Within a Cartesian coordinate system, the orientation vector $\vec{p}$ can be properly defined using two angular values [θ, α] [48]:

(1) $$\vec{p}=p_{i}\vec{e1}+p_{j}\vec{e1}+p_{k}\vec{e3}=\left(cos\alpha \;\cdot \;cos\theta \right)\vec{e1}+\left(cos\alpha \;\cdot \;sin\theta \right)\vec{e2}+\left(sin\alpha \right)\vec{e3}.$$

The probability distribution function $\psi (\vec{p})$ can be used to address the orientation of all fibers within the region [48]:

(2) $$\psi \left(\vec{p}\right)=\psi \left(\theta ,\alpha \right),\theta \in \left(0,\pi \right),\alpha \in \left(-\frac{\pi }{2},\frac{\pi }{2}\right),$$

such that the fiber distributions with a specific region ω can be statistically described via a second-order orientation *a*, which is derived from the probabilities:

(3) $$a=\left[\begin{array}{ccc} a_{11} & a_{12} & a_{13}\\ \; & a_{22} & a_{23}\\ sym & \; & a_{33} \end{array}\right],a_{ij}=\int_{\Omega }p_{i}p_{j}\psi \left(\vec{p}\right)d\vec{p}.$$

The tensor *a* possesses properties of symmetry with $a_{ij}=a_{ji}$, and it holds a trace equal to 1. And the orientation tensor can be further decomposed into a diagonal tensor Λ and a rotation one *R*:

(4) $$a=R\left(\gamma_{1},\gamma_{2},\gamma_{3}\right)\Lambda R^{T}\left(\gamma_{1},\gamma_{2},\gamma_{3}\right),$$

where $\Lambda =\left[\begin{array}{ccc} a_{1} & & \\ & a_{2} & \\ & & a_{3} \end{array}\right]$, $\gamma_{1}$, $\gamma_{2}$ and $\gamma_{3}$ are rotation angles around axis of e1, e2 and e3, respectively.

#### 2.1.2. The Two-Step Homogenization Method

**Figure 2:**
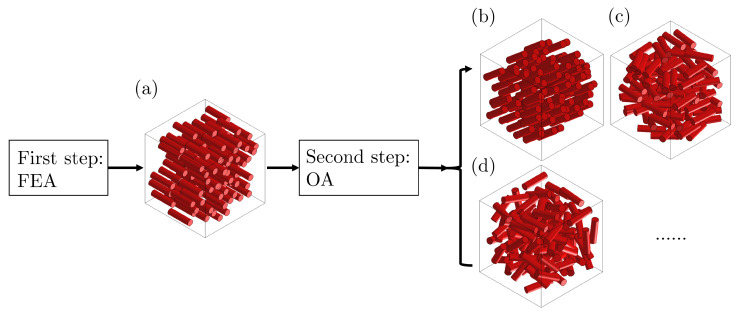
Illustration of the two-step homogenization method: calculation of homogenized mechanical properties of RVEs considering. (**a**) unidirectional fiber distribution in two dimensions; (**b**) unidirectional fiber distribution in three dimensions; (**c**) random fiber distributions in two dimensions; (**d**) random fiber distributions in three dimensions.

The process of the two-step homogenization method is illustrated in Figure 2. Composites having aligned short fibers with uniform length and mechanical properties are first considered to build the unidirectional RVEs (noted as UD RVEs). Accordingly, their homogenized properties can be obtained via EF simulations. Afterward, OA is applied to UD RVEs with orientation tensors to calculate the properties of composite RVEs with distributions of fiber orientation, without modeling of RVEs with realistically distributed fibers [3,9].

Derivation of the stiffness tensor can be obtained as [13]:

(5) $$\begin{array}{cc} C_{ijkl}= & B_{1}\left(a_{ijkl}\right)+B_{2}\left(a_{ij}\delta_{kl}+a_{kl}\delta_{ij}\right)+B_{3}\left(a_{ik}\delta_{jl}+a_{il}\delta_{jk}+a_{jl}\delta_{ik}+a_{jk}\delta_{il}\right)\\ \; & +B_{4}\left(\delta_{ij}\times \delta_{kl}\right)+B_{5}\left(\delta_{ik}\times \delta_{jl}+\delta_{il}\times \delta_{jk}\right) \end{array}$$

(6) $$\begin{array}{cc} B_{1} & =C_{11}^{UD}+C_{22}^{UD}-2C_{12}^{UD}-4C_{66}^{UD}\\ B_{2} & =C_{12}^{UD}-C_{23}^{UD}\\ B_{3} & =C_{66}^{UD}+\frac{1}{2}\left(C_{23}^{UD}-C_{22}^{UD}\right)\\ B_{4} & =C_{23}^{UD},\\ B_{5} & =\frac{1}{2}\left(C_{22}^{UD}-C_{23}^{UD}\right), \end{array}$$

where $C_{ijkl}$ represents the stiffness matrix of the desired arbitrary orientation RVE, $a_{ij}$ and $a_{ijkl}$ denote its second-order and fourth-order orientation tensors respectively, and $\delta_{ij}$ is the Kronecker delta. $B_{i}\left(i=1-5\right)$ in Equation (6) are parameters that are derived from the stiffness matrix of UD RVE $C_{ijkl}^{UD}$.

The 21 components of the stiffness matrix *C* can be acquired from Equations (5) and (6):

(7) $$C=\left[\begin{array}{cccccc} C_{11} & C_{12} & C_{13} & C_{14} & C_{15} & C_{16}\\ \; & C_{22} & C_{23} & C_{24} & C_{25} & C_{26}\\ \; & \; & C_{33} & C_{34} & C_{35} & C_{36}\\ \; & \; & & C_{44} & C_{45} & C_{46}\\ \; & sym & \; & & C_{55} & C_{56}\\ \; & \; & & \; & & C_{66} \end{array}\right].$$

### 2.2. Feature Selection and Data Preparation

#### 2.2.1. Feature Selection

The homogenized mechanical properties of SFRPCs are mainly determined by the properties of matrix and fiber, as well as the microstructure parameter of fibers [2]. For this specific problem, we intend to make the prediction of the composite matrix using 12 features, with the selection based on the strategies discussed in [7,14].

The 12 input features include 7 properties [$E_{m}$, $\nu_{m}$, $E_{f}$, $\nu_{f}$, *d*, *a*, VF], and 5 individual components of the orientation tensor in Equation (3) for indicating the distribution of the short fibers. *E* and ν denote Young’s modulus and Poisson’s ratio, respectively, with the subscript *m* and *f* representing the matrix and fiber. *d*, *a*, and VF describe the fiber diameter, the ratio of fiber length to diameter, and the volumetric fraction of fibers.

#### 2.2.2. Data Preparation

The dataset is constructed by numerical simulations using composite RVEs with the two-step homogenization method.

**Figure 3:**
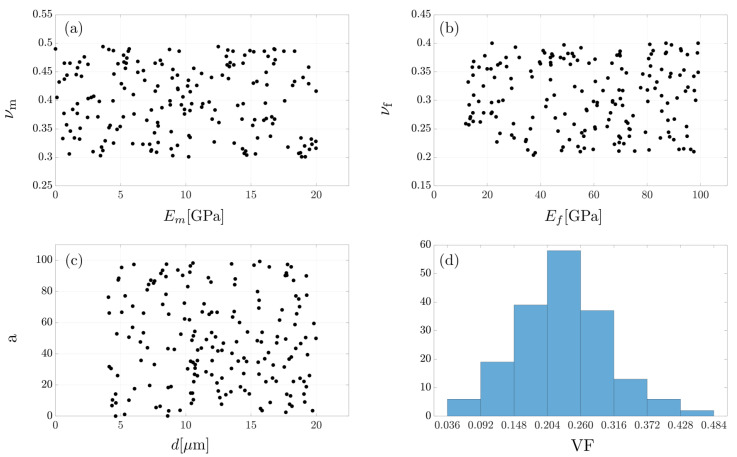
Parameter pairs for UD RVE simulations: (**a**) elastic properties of $E_{m}$ and $\nu_{m}$; (**b**) elastic properties of $E_{f}$ and $\nu_{f}$; (**c**) fiber diameter *d* and aspect ratio *a*; (**d**) fiber volume fraction VF.

Firstly, 180 sets of UD RVEs are set up for parametric simulations in ABAQUS 6.13. The parameter space in UD RVE simulations is designed as in Figure 3. The parameter pairs for matrix Young’s modulus and Poisson’s ratio (Figure 3a), fiber Young’s modulus and Poisson’s ratio (Figure 3b), fiber diameter and fiber aspect ratio (Figure 3c), and fiber volume fraction and diameter (Figure 3d) are chosen [14]. The fiber volume fraction VF follows a normal distribution and the rest of the parameter pairs obey uniform distributions.

Secondly, for each UD RVE, 60 different orientation tensors are applied using the OA method, yielding a total of 10,800 sets for calculation of the homogenized mechanical properties. The uniformity of orientation distributions is explored by creating diagonal tensors initially and then employing the rotation angles.

In particular, we choose ten diagonal tensors, three of which represent unidirectional distribution ($a=\left[\begin{array}{ccc} 1 & & \\ & 0 & \\ & & 0 \end{array}\right]$), two-dimensional random distribution ($a=\left[\begin{array}{ccc} 0.5 & & \\ & 0.5 & \\ & & 0 \end{array}\right]$), and three-dimensional isotropic distribution ($a=\left[\begin{array}{ccc} 0.33 & & \\ & 0.33 & \\ & & 0.33 \end{array}\right]$). The remaining seven tensors symbolize randomly distributed fibers in three-dimensional space. Thus, for each set of UD RVEs, 60 different orientation tensors can be obtained by randomly rotating each diagonal tensor five times. Additionally, to prevent excessive concentration of angles uniform distribution is applied to $\gamma_{1}$ and $\gamma_{3}$ in the interval (0,π), and the following formula is used to sample $\gamma_{2}$:

(8) $$t=(\gamma_{2}-sin\left(\gamma_{2}\right))/\pi ,0<t<1,$$

where *t* is selected randomly in interval (0,1).

**Figure 4:**
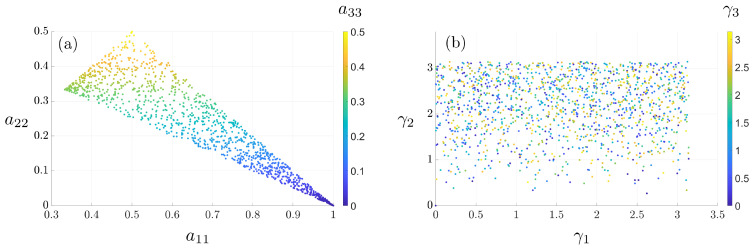
Distributions of: (**a**) diagonal tensor $a_{11},a_{22},a_{33}$; (**b**) rotation angles of $\gamma_{1},\gamma_{2},\gamma_{3}$ used to rotate diagonal orientation tensors.

Figure 4 displays the distribution of diagonal tensor and rotation angles, created according to the process explained above. In Figure 4a, greater scatterings of data points are seen at the triangular surface’s vertices, particularly in the case of a planar or 3D-random surface. This is a result of the limitations of the parameter space and the random generation of fiber orientation sets as in Equation (8). Figure 4b shows the randomness of the first 2000 sets of angles. Three angles in each set are used to rotate the diagonal orientation tensors around three axes. It can be found that there are no significant gaps or clusters of data points in the distribution.

#### 2.2.3. Data Analysis

**Figure A1:**
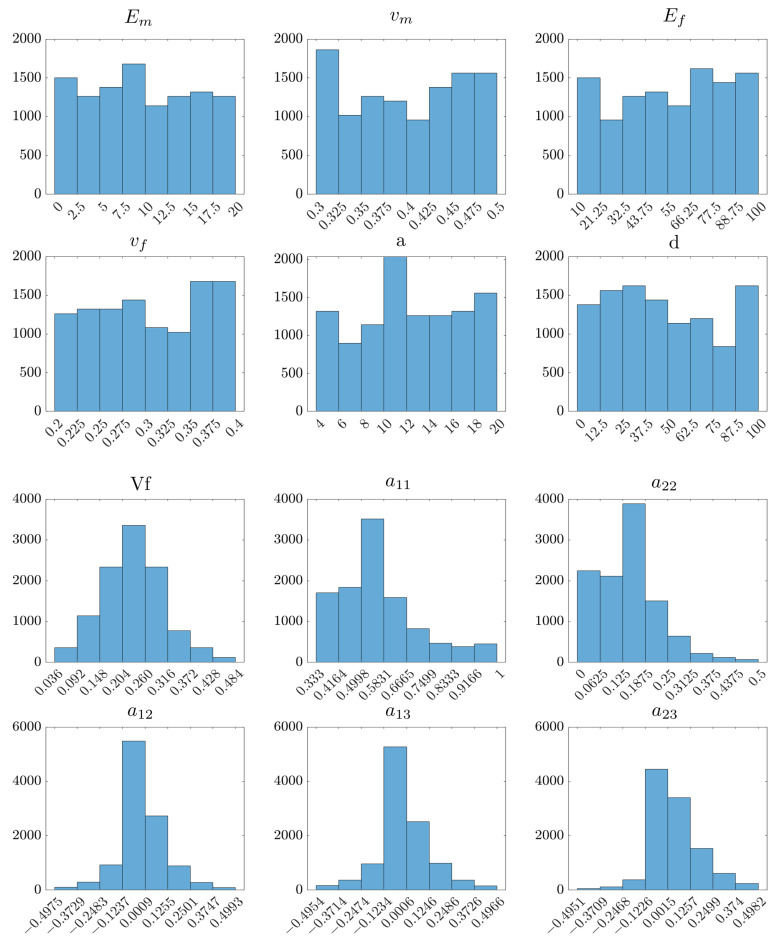
Distribution of input features for 10,800 samples in the dataset.

**Figure A2:**
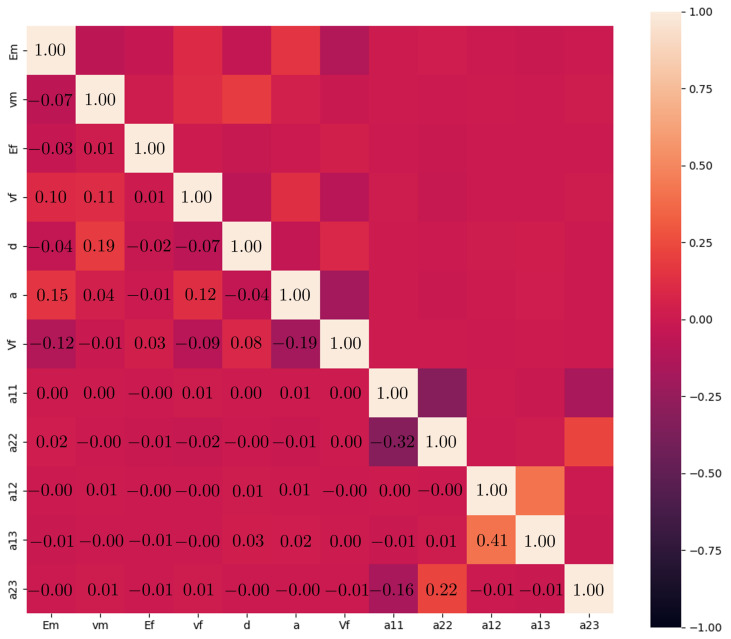
Correlation matrix analysis of the input features in the dataset via PCC measurement. The PCC has a number between –1 and 1 that measures the strength and direction of the relationship between two variables [36].

The dataset with 10,800 samples is collected by the hybrid simulation framework using the two-step homogenization algorithm. Figure 3 and Figure 4 visualize the distribution of the input features following the statements in [14]. Figure A1 in Appendix A further describes the distribution of each parameter, and Figure A2 shows the correlation matrix that is obtained using a correlation study analysis.

The correlation analysis is presented via the criterion based on the Pearson Correlation Coefficient (PCC) [49], which is a measure of the linear dependence between two random variables. The PCC has a number between –1 and 1 that measures the strength and direction of the relationship between two variables. As noted in Figure A2, the input features are basically independent, while the tensor variables of $a_{11}$ and $a_{22}$ in Figure 4 implying negative but still weak correlations. This is aligned with the derivation of the diagonal tensor components in the two-step homogenization method, and the understanding of the mechanical definition in the knowledge about fiber-reinforced polymer composites [50].

However, as stated in [51], PCC cannot reflect a certain correlation with each other. It is therefore necessary to apply the optimal ML model combined with the ML explainer based on SHAP to investigate the internal relations between model outputs and input features.

More knowledge and mathematical theories for PCC can be found in [36].

### 2.3. Ensemble Machine Learning

This study uses a stacking ensemble model [37,38], which involves fitting numerous model types to the same data and using a different model to learn how to combine the predictions most effectively.

#### 2.3.1. Base Learners

Enlightened by reference works that are devoted to the homogenization problem within composites that are reinforced with different fiber types in [12,14,22,23,24,25,28,37,52], as we discussed in the introduction section, we have experimented with a variety of base models based on the dataset, ranging from the basic SVR, RF, and DT models to more complicated ET, XGBoost, and LGBM models.

The preliminary comparison investigation also found that the basic yet interpretable model does not perform well on this challenge ($R^{2}$ values less than 0.85), given the distribution of short fibers introduces more features that influence the anisotropy of the composites. This is consistent with the findings of earlier studies that used simulated datasets to predict composite homogenization. We therefore subsequently decided on three options for designing the EML model: ET, XGBoost, and LGBM.

In particular, the ET algorithm is an enhancement of the Random Forest algorithm that employs a “Bagging” technique and consists of numerous decision tree learners [53,54]. Both XGBoost and LGBM use a gradient-boosting framework. LGBM performs leaf-wise (vertical) growth as opposed to level-wise (horizontal) growth in XGBoost, which produces more loss reduction and, as a result, improved accuracy while being faster [55]. As such, those models are trained on the unchanged train dataset, ensuring that they make different assumptions and, in turn, have less correlated prediction errors.

It should be noted that although the base learners are complicated models, they are far simpler than neural network-based models. Appendix B summarizes the preliminary knowledge for the base learners, such as backgrounds, objective functions, and loss functions. In terms of models such as SVR, RF, and DT models, which could have been learned in-depth in Reference [36], the basic introductory knowledge will not be addressed in this work.

Furthermore, functional interfaces for multiple classification, regression, and clustering methods are available in the machine learning library Scikit-learn [56]. The three base learners and models of SVR, RF, and DT are also included. Users can simply utilize the library to create models using the Python programming language after they have a basic understanding of the basic mathematical concepts.

#### 2.3.2. Stacking Mode

**Figure 5:**
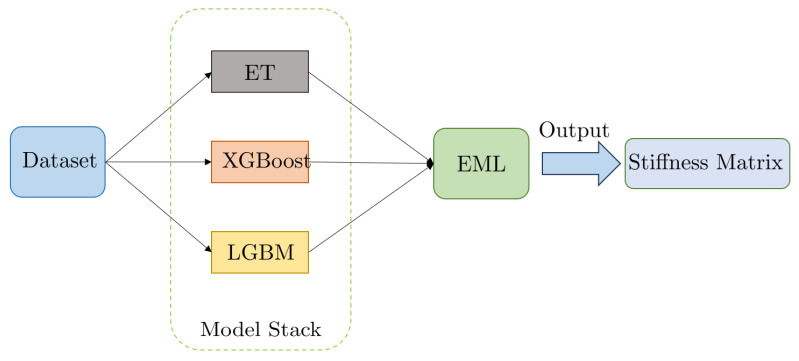
Illustration of the stacking algorithm.

As shown in Figure 5, the stacking model takes into account diverse base learners, trains them concurrently, and then combines them by training a meta-learner to produce a prediction based on the predictions of the various base learners.

The base learners are trained using the original dataset, in which the 5-fold cross-validation is employed to avoid overfitting. Each base learner has different hyperparameters that need to be optimized. An ensemble learning model inputs the predictions as the features and the target is the ground-truth values in the dataset, it attempts to learn how to best combine the input predictions to make a better output prediction [36].

This work trains the EML model on the constructed dataset to predict the 21 components of the stiffness matrix based on the 12 input features.

### 2.4. The SHAP-Based Interpretation Analysis

Low interpretability is often caused by machine learning models’ complexity and highly non-linear architecture. Compared to conventional approaches that just specify what feature is crucial but fail to explain how that feature influences the predictions [57,58], the SHAP method offers a more effective means for evaluating the “black box” of machine learning models [41]. Based on cooperative game theory, the method uses SHAP values to quantify the impact of individual features by calculating the contribution among various permutations of feature combinations.

The explanatory model f(x) in response to a input feature *x* can be expressed as [41]:

(9) $$f\left(x\right)=\varphi_{0}+\sum_{i=1}^{N}\varphi_{i}x_{i}^{\prime },$$

where $\varphi_{0}$ is a constant of “ base value” depicting the average of all predicted values, and *N* is the total number of input features, and $x_{i}^{\prime }$ is a binary feature value where $x_{i}^{\prime }=1$ represents a present feature and $x_{i}^{\prime }=0$ represents absent features. $\varphi_{i}$ is the SHAP value of the *i*-th feature that measures the contribution of the *i*-th feature from a composition of all features.

The SHAP analysis is compatible with various model types. In SHAP, each feature is assigned an importance value, and the influence of all the features on the predicted responses can be assessed via global and local interpretations. According to Equation (9), the global interpretation uses SHAP values to depict feature relevance with a positive or negative impact on predictions, whereas the local interpretation generates SHAP values and illustrates how the fed-in features contribute to that specific prediction [32,44].

### 2.5. Hyperparameter Optimization and Model Training

We build, train, and optimize the EML model with the PyTorch package in Python [59].

The model hyperparameter adjusting performs a crucial factor in the prediction performance of the ML model. Selecting the optimal values for the hyperparameters of a machine learning algorithm is a critical step for developing an accurate and trustworthy model [60]. This work implements hyperparameters optimization, using the grid search method [61,62], to detect all possible values in the search space in order to find the optimal structure for the three base learners. Additionally, 5-fold cross-validation is used to overcome the issue of overfitting and is combined with a hyperparameter tuning technique based on grid search to identify the optimal values.

Basic knowledge of ML techniques of grid search and K-fold cross-validation are provided in Appendix C.1 and Appendix C.2, respectively. For the three base learners, each algorithm had its unique hyperparameter to be optimized, with a detailed description provided in Appendix C.3.

The optimal values of hyperparameters of the three base learners are listed in Table 1.

## 3. Results and Discussion

### 3.1. Validation of Dataset Based on the Two-Step Homogenization Method

The method utilizes a statistical tensor to effectively involve the fiber orientations in homogenization calculations [2,14,63]. A brief validation analysis is first conducted to ensure the accuracy of the two-step homogenization method.

**Figure 6:**
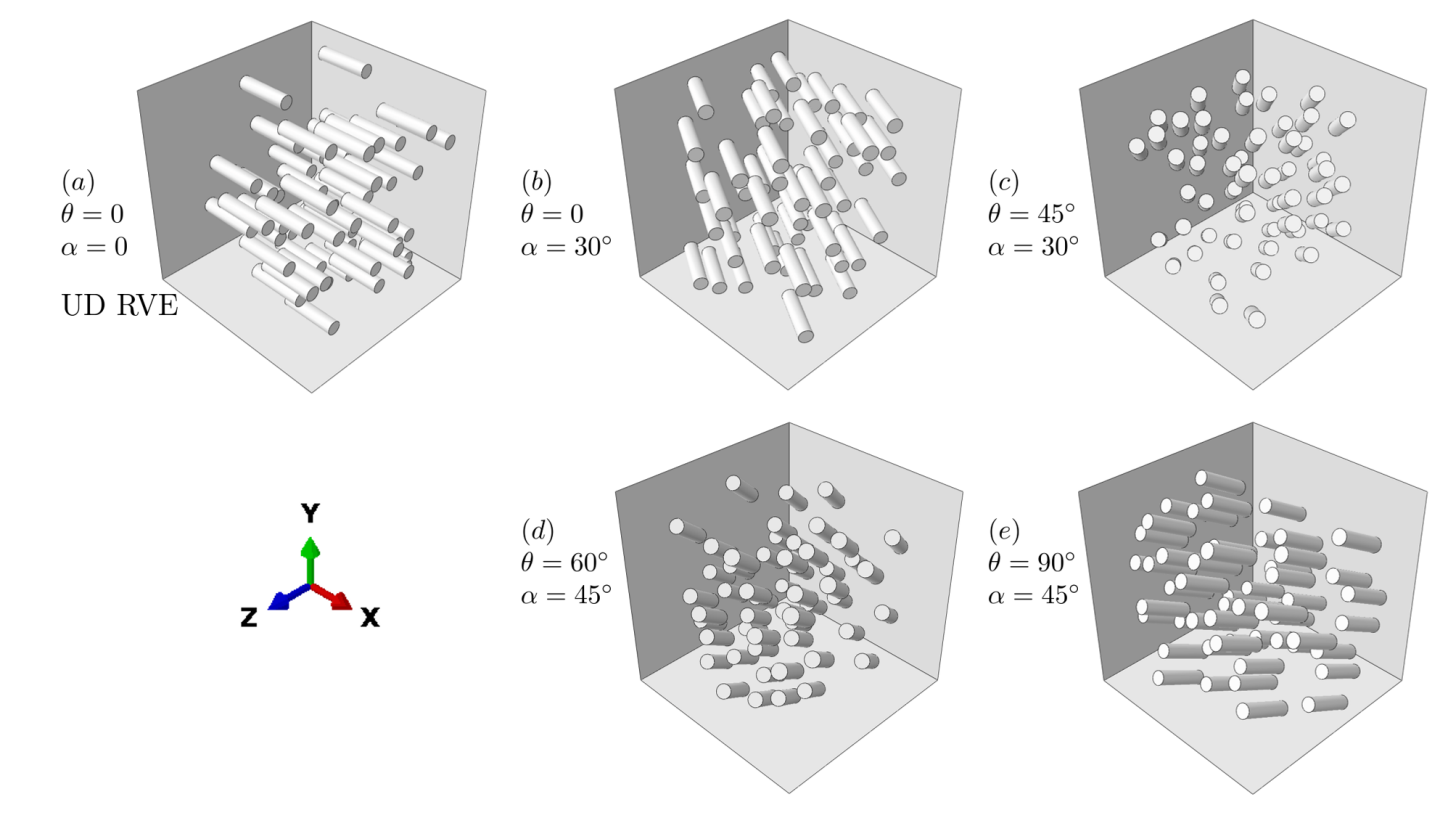
Validation of the numerical dataset with RVEs in ABAQUS with designed fiber orientations of: (**a**) UD RVE(θ=0,α=0); (**b**) $\theta =0,\alpha =30^{\circ }$; (**c**) $\theta =45^{\circ },\alpha =30^{\circ }$; (**d**) $\theta =60^{\circ },\alpha =45^{\circ }$; (**e**) $\theta =90^{\circ },\alpha =45^{\circ }$.

Figure 6 demonstrates one UD RVE and four RVEs with different fiber rotation angles. The microstructural parameters for UD RVE (specified as α=0,β=0) in Figure 6a and the simulated homogenization mechanical properties are given Table 2 and Table 3, respectively.

The homogenized mechanical properties for RVEs in Figure 6b–e are first calculated using the two-step homogenization method. Meanwhile, full FEA simulation results using the RVEs are conducted in ABAQUS to verify the above hybrid approach, with the comparison shown in Table 4.

It can be seen that, compared to the FEA-determined results, the average error of the calculated results using the two-step homogenization method is 0.82%. Hence, the homogenized mechanical properties of the SFRPC RVEs with the considered fiber orientations derived by the two-step homogenization framework are validated as reliable and effective.

### 3.2. Model Evaluation on Prediction Accuracy

#### 3.2.1. Evaluation Metrics

The coefficient of determination $R^{2}$, mean squared error (MSE), and mean absolute percentage error (MAPE) are adopted as evaluation metrics, with the following formulas [64]:

(10) $$R^{2}=1-\frac{\sum {\left({\hat{y}}_{i}-y_{i}\right)}^{2}}{\sum {\left(\bar{y}-y_{i}\right)}^{2}}$$

(11) $$MSE=\frac{1}{n}\sum_{n}^{i=1}{\left(\hat{y_{i}}-y_{i}\right)}^{2}$$

(12) $$MAPE=\frac{100\%}{n}\sum_{i=1}^{n}\left|\begin{array}{c} \frac{\hat{y_{i}}-y_{i}}{y_{i}} \end{array}\right|$$

where $y_{i}$ is the ground-truth of the *i*-th sample point in the database, ${\hat{y}}_{i}$ is the predicted values by the EML model, $\bar{y}$ is the average value of all sample, and *n* is the number of sampels.

#### 3.2.2. Accuracy Comparison between the EML and Base Learners

The EML model combines three basic learners to make predictions of the matrix with components from 12 input features. Table 5 summarizes the comparison of the prediction accuracy of the EML model with three base learners on both the training and testing set. On an overall basis, the model’s accuracy on the training dataset is higher. Among the base learners, the LGBM model achieves the highest $R^{2}$ values of 0.984 and 0.946 on the train and test datasets, respectively. It also performs the best in the two other evaluation metrics of MSE and MAPE. The three base learners are outperformed by the EML model in each evaluation metric ($R^{2}=0.988$, $MSE=2.545\times 10^{6}$, MAPE=0.567% on train, and $R^{2}=0.952$, $MSE=1.260\times 10^{16}$, MAPE=0.906% on test), indicating an effective enhancement of the prediction accuracy.

**Figure 7:**
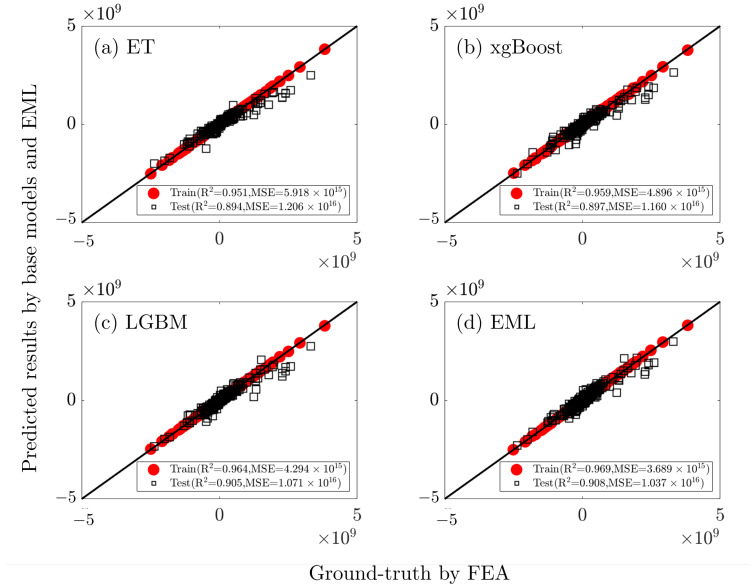
Scatter plots of $R^{2}$ of $C_{15}$ between the FEA-determined ground truth values and the predictions from models of ET, XGBoost, LGBM, and the EML.

Figure 7 further visualizes the computed $R^{2}$ with the scatter plots of predicted vs. ground truth values of matrix component $C_{15}$ using different machine learning models. It can be seen that the ET model is the least accurate predictor of the three base learners, with $R^{2}$ values on the train and test sets of 0.951 and 0.894, respectively. In contrast, the $R^{2}$ value for the EML model on the train and test sets, respectively, is promoted to 0.969 and 0.908, making it the best fitter.

#### 3.2.3. Model Performance of the EML Model on a Testing Sample

To fully evaluate the model’s efficacy in making predictions on the 21 components of matrix *C*, Table 6 summarizes the evaluation metrics of $R^{2}$, MSE, and MAPE of the EML model’s outputs on a testing sample. It can be observed that the EML model predicts 9 features with high precision, such as $C_{11}$, with a $R^{2}$ value greater than 0.999. The prediction accuracy is slightly reduced but still reaches a high level of $R^{2}>0.90$ for the remaining 12 outputs, such as $C_{14}$, maintaining the appearance of a well-fitted model.

The variation in prediction accuracy is commonly seen in developing data-driven models, which is attributed to the dataset construction, and the selection of input features [37]. Overall, the EML model showcases superior accuracy in terms of using 12 fed-in features and predicting the matrix of the polymer composites.

### 3.3. Model Interpretation via SHAP Analysis

In order to provide a comprehensive overview of how anisotropic mechanical properties are influenced by microstructural variables, the original output stiffness matrix *C* in Equation (7) has been translated into homogenized Young’s modulus *E*, Poisson’s ratio ν, and Shear modulus *G*. Most concerned Young’s modulus $E_{11}$, $E_{22}$, and $E_{33}$, which characterize the anisotropy of SFRPCs, are specifically subjected to global and local SHAP analysis [11].

#### 3.3.1. Global Interpretation

**Figure 8:**
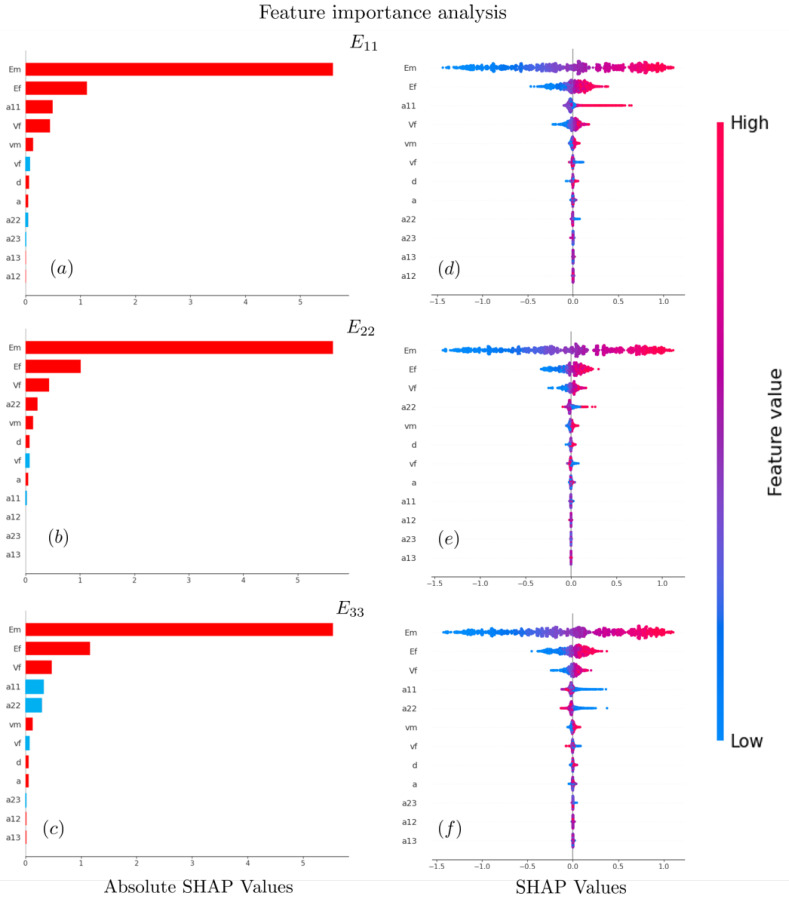
The global interpretation of feature importance analysis for Young’s modulus of $E_{11}$ in (**a**,**d**), $E_{22}$ in (**b**,**e**), and $E_{33}$ in (**c**,**f**). Bar plots in (**a**–**c**) rank the order of feature importance with the color code of red indicating positive impact and blue implying negative impact. Beeswarm plots in (**d**–**f**) highlight the significance of the feature in relation to predicted outcomes.

Figure 8 presents a global interpretation of feature importance analysis for $E_{11}$, $E_{22}$, and $E_{33}$. Figure 8a–c are bar plots of SHAP values, which illustrate the importance rank, and direction of each input feature. As shown, the homogenized Young’s modulus is primarily influenced by three factors: the Young’s modulus of $E_{m}$, $E_{f}$, and the fiber content VF. Notably, all of these factors exert a positive influence on the homogenized Young’s modulus. This phenomenon arises from the fact that $E_{m}$ and $E_{f}$ represent vital mechanical properties of composite material constituents, while VF governs the proportion of strengthening components. In general, enhancing both the mechanical properties of constituents and increasing the content of reinforcement components contributes to the improved homogenization performance of the composite material.

The orientation tensor components $a_{11}$, $a_{22}$, and $a_{33}$ play a pivotal role in determining the anisotropy of composites. It can be observed that Young’s modulus $E_{11}$ in Figure 8 is markedly and positively impacted by the feature $a_{11}$. This occurs because $a_{11}$ characterizes the distribution of fiber orientation in the 1-direction, with larger values of $a_{11}$ indicating a greater presence of fibers aligned in the 1-direction. Similar findings show that the features defining fiber orientation $a_{22}$ and $a_{33}$ appropriately determine anisotropy for $E_{22}$ in Figure 8b and $E_{33}$ in Figure 8c. It is worth noting that, in Figure 8c, $a_{11}$ and $a_{22}$ display negative influences on $E_{33}$, which attributes to the relation between the diagonal tensor of $a_{33}=1-a_{11}-a_{22}$, as described in Figure 4.

Figure 8d–f are represented as beeswarm plots that highlight the significance of the feature in relation to the actual relationships with the predicted outcomes. The beeswarm plots reveal the same feature information as the bar plots. The horizontal position of the dot is determined by the SHAP value of that feature, and the dots “pile up” along each feature row to rank the feature’s importance. The color code represents the original numerical value of a feature. For example, the high value of $E_{m}$ and $E_{f}$ will increase the value of Young’s modulus in each direction.

Both the bar plots and beeswarm plots indicate that the other features, including Poisson’s ratio, fiber length-diameter ratio, and the orientation tensor’s deviatoric components, have been identified to support the predictions while having fewer influencing effects than the other aspects.

The SHAP-based explanation is consistent with theories and knowledge from mechanism analysis in simulations [11] and experimental findings [46].

#### 3.3.2. Local Interpretation

The goal of SHAP local interpretability is to describe the contributions made by each input feature in terms of how individual predictions are made.

**Figure 9:**
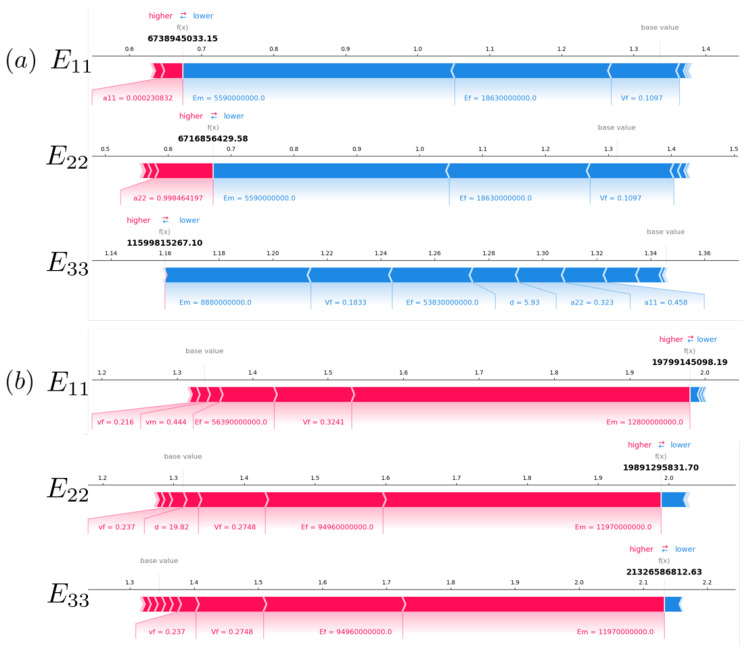
Local SHAP analysis for the $E_{11}$, $E_{22}$, and $E_{33}$ predictions of two testing samples: (**a**) Sample a and (**b**) Sample b.

We select two testing samples, and Figure 9 visualizes the positive and negative contributors in predicting $E_{11}$, $E_{22}$, and $E_{33}$. The red arrows indicate a positive impact of the corresponding input feature on the output, while the blue arrows signify a negative impact. According to Equation (9), the length of the arrows reflects how much the input feature has influenced the model.

For Sample (a), the small values of $E_{m}=5.590\;\mathrm{GPa},E_{f}=18.630\;\mathrm{GPa},VF=0.110$ show negative impact and results in comparable small magnitudes of $E_{11}$, $E_{22}$, and $E_{33}$. In comparison, the diagonal tensor component of $a_{11}$, $a_{22}$, and $a_{33}$ accordingly promotes Young’s modulus which signifies a positive impact.

Sample (b) contains a scenario that is the opposite of Sample (a). For Sample (b), the local analysis quantifies the large inputs of $E_{m}=11.970\;\mathrm{GPa},E_{f}=94.960\;\mathrm{GPa},VF=0.275$, leading to rather small magnitudes of $E_{11}$, $E_{22}$, and $E_{33}$. By contrast, features that denote the fiber orientation ($a_{11}$, $a_{22}$, and $a_{22}$) show negative impacts.

It can be found that the local analysis’ findings are consistent with the global interpretations, while it details the modulating mechanism of the influencing features in each individual case. Understanding the feature contributions and their underlying modulating mechanism can also provide an optimized strategy to customize the composite properties by following the local analysis [65].

### 3.4. Model Generalization on Experimental Data

On the prepared simulation dataset, the trained EML model exhibits excellent accuracy and efficiency with the predictions being interpretable. To determine the model’s generalizability, we further apply the EML model to the unknown dataset obtained from experiments.

The polymer composite, known as PA15 due to 15% fiber weight fractions orginiates from [46], is a composite made of glass fibers and a polymer matrix. Another set of experimental data is derived from [47], and the material is referred to as PA6GF35, a polyamide-6 matrix with 35% (by weight) glass-fiber-reinforcement. Table 7 lists the microstructural parameters of the two sets of polymer composites, including the elastic properties of matrix and fiber, as well as the measured fiber orientation tensor. Using the given parameters, we employ the EML model to predict the macroscopic mechanical properties of those short fiber-reinforced composites.

**Figure 10:**
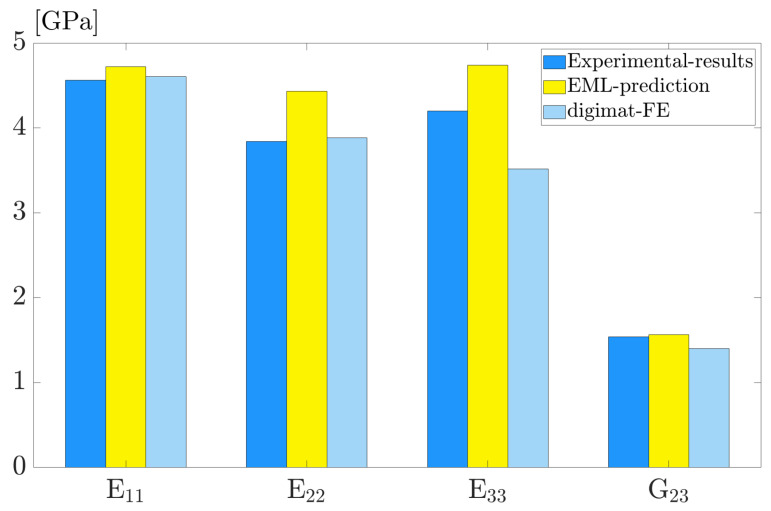
Model generalization on PA15: comparison between EML predictions with experimental results and Digimat-FE calculations.

For PA15, Figure 10 compares the experimental measurements of $E_{11}$, $E_{22}$, $E_{33}$ and $G_{23}$, with the predictions from the EML model, and FEA results using Digimat software. The proposed EML model performs well in predicting elastic properties, with a maximum relative error of less than 10%.

**Figure 11:**
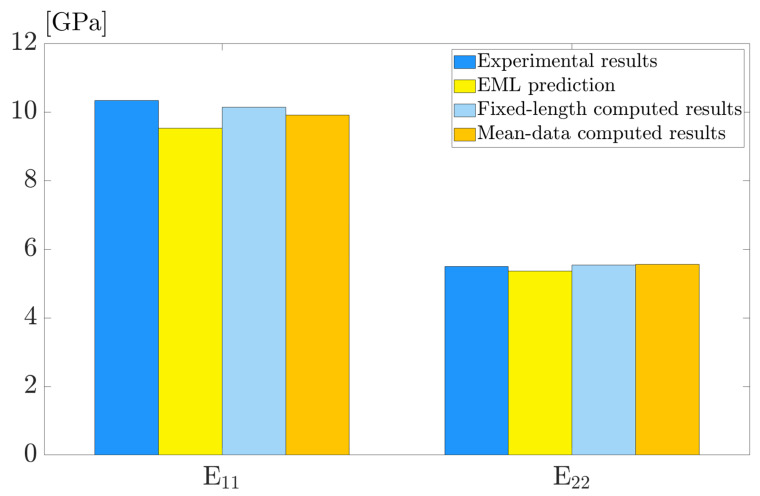
Model generalization on PA6GF35: comparison between EML predictions with experimental results and two high-fidelity computational models.

For PA6GF35, Figure 11 illustrates a comparison between the experimental results of Young’s modulus $E_{11}$ and $E_{22}$ and the EML-predicted results. Meanwhile, two high-fidelity computational models reported from [47] are included for prediction comparison, namely the “Mean data” and “Fixed length”. It can be noted that the EML model agrees well with the experimental results with a maximum error of less than 3%, with the predicted $E_{11}$ and $E_{22}$ closely aligned with the high-fidelity models.

In terms of prediction efficiency, as shown in Table 8, using a mesh of 19,937 elements, the Digital-FE requires 2911 s to complete relevant calculations. In contrast, the EML model significantly lowers the cost of computing and produces predictions in less than one second.

The EML technique displays a highly generalized machine learning model inside the two experimental scenarios with polymer composite reinforced with various fillings of glass fibers. Furthermore, it has been demonstrated to be significantly more cost-effective than high-fidelity computational models while maintaining excellent accuracy.

## 4. Conclusions and Outlook

This study proposes a novel interpretable and high-generalizability ensemble learning model to efficiently predict the homogenized properties of SFRPCs. A micromechanical model of a two-step homogenization algorithm is integrated to construct a diverse and representative dataset, in which the random-distributed fibers are efficiently implemented. We perform hyperparameter optimization for each base learner of ET, XGBboost, and LGBM. The EML model is comprehensively assessed using metrics of accuracy, efficiency, interpretability, and generalization capabilities. The following conclusions can be drawn:

(1) The two-step homogenization algorithm is validated as an effective approach with which to consider different fiber orientations, which favors the creation of a trustworthy dataset with a variety of the chosen features.
(2) The EML model outperforms the base members of ET, XGBoost, and LBGM on prediction accuracy, achieving $R^{2}$ values of 0.988 and 0.952 on the train and test datasets, respectively.
(3) According to the SHAP global analysis, the homogenized elastic properties are significantly influenced by $E_{m}$, $E_{f}$, and VF, whereas the anisotropy is predominantly determined by $a_{11}$, $a_{22}$, and $a_{33}$. The SHAP local interpretation distinguishes the key modulating mechanism between the key features for individual predictions.
(4) The EML algorithm showcases a highly generalized machine learning model on experimental data, and it is more efficient than high-fidelity computational models by drastically reducing computational expenses and preserving high accuracy.

The paradigm proposed in this study holds potential applications to customize the fiber arrangements with a different filling of fibers in polymer structure and, as a result, offers manufacturing process optimization techniques. The model is first evaluated for generalizability on the basis of two experimental cases. It can be further packaged as a Graphical User Interface (GUI) in order to improve its practical utility [66,67] on the topic of analyzing homogenized polymer composites with short fibers.

The limitations are also acknowledged. Current work focuses on analyzing the elastic properties of the SFRPCs by considering one type of reinforcing fiber in the polymer structure. The two-step homogenization method utilizes a statistical tensor to effectively involve the fiber orientations in homogenization calculations, while the cross-linking of fibers, the mechanical interactions, and the morphology of fiber curvatures are ignored [68,69]. Hybrid composites contain more than two types of fiber fillings in polymer composites [70], mechanical interactions between fibers and polymer matrix, matrix defects, voids, and failure should be considered in future investigations [71].

## Figures and Tables

**Table 1:** The optimal hyperparameters for the base learner models using grid search.

Base Learner Model	Hyperparameters	Optimal Values
ET	n_estimators	160
	max_depth	21
	min_samples_split	2
	min_samples_leaf	1
	max_features	11
XGBoost	n_estimators	200
	learning_rate	0.15
	max_depth	6
	min_child_weight	2
	colsample_bytree	1
	subsample	1
	gamma	0
LGBM	n_estimators	360
	learning_rate	0.22
	max_depth	−1
	min_child_weight	3
	colsample_bytree	1
	subsample	0.7
	gamma	0
	num_leaves	31

**Table 2:** The microstructural parameters for the UD RVE in Figure 6a.

Parameter	$E_{m}$[GPa]	$\nu_{m}$	$E_{f}$[GPa]	$\nu_{f}$	Fiber Diameter [μm]	Fiber Length [μm]	VF
Magnitude	1.6	0.4	69	0.15	2	8	5%

**Table 3:** The simulated homogenized mechanical properties for the UD RVE in Figure 6a.

Mechanical Properteis	$E_{11}$[GPa]	$E_{22}$[GPa]	$E_{33}$[GPa]	$\nu_{12}$	$\nu_{13}$	$\nu_{21}$
Magnitude	2.187	1.827	1.830	0.393	0.392	0.328
**Mechanical Properteis**	**$G_{23}$[GPa]**	**$G_{13}$[GPa]**	**$G_{12}$[GPa]**	** $\nu_{23}$ **	** $\nu_{31}$ **	** $\nu_{32}$ **
Magnitude	0.629	0.637	0.637	0.441	0.328	0.441

**Table 4:** Validation of simulations obtained via the two-step homogenization method and full FEA simulations on composite RVEs with different fiber orientations.

$\theta =0,\alpha =30^{\circ }$	$E_{11}$ [GPa]	$E_{22}$ [GPa]	$E_{33}$ [GPa]	$\nu_{12}$	$\nu_{13}$	$\nu_{21}$
FEA	2.009	1.871	1.806	0.369	0.427	0.344
Two-step	1.929	1.827	1.776	0.376	0.421	0.356
Relative error	3.95%	2.31%	1.69%	1.84%	1.46%	3.57%
	** $\nu_{23}$ **	** $\nu_{31}$ **	** $\nu_{32}$ **	**$G_{23}$ [GPa]**	**$G_{13}$ [GPa]**	**$G_{12}$ [GPa]**
FEA	0.415	0.384	0.400	0.643	0.719	0.645
Two-step	0.413	0.387	0.401	0.634	0.706	0.636
Relative error	0.45%	0.85%	0.18%	1.36%	1.74%	1.32%
** $\theta =45^{\circ },\alpha =30^{\circ }$ **	**$E_{11}$ [GPa]**	**$E_{22}$ [GPa]**	**$E_{33}$ [GPa]**	** $\nu_{12}$ **	** $\nu_{13}$ **	** $\nu_{21}$ **
FEA	1.786	1.786	1.777	0.398	0.396	0.398
Two-step	1.780	1.780	1.775	0.398	0.395	0.398
Relative error	0.30%	0.35%	0.09%	0.05%	0.19%	0.01%
	** $\nu_{23}$ **	** $\nu_{31}$ **	** $\nu_{32}$ **	**$G_{23}$ [GPa]**	**$G_{13}$ [GPa]**	**$G_{12}$ [GPa]**
FEA	0.396	0.394	0.394	0.666	0.666	0.682
Two-step	0.395	0.395	0.395	0.668	0.668	0.686
Relative error	0.14%	0.16%	0.28%	0.32%	0.36%	0.59%
** $\theta =60^{\circ },\alpha =45^{\circ }$ **	**$E_{11}$ [GPa]**	**$E_{22}$ [GPa]**	**$E_{33}$ [GPa]**	** $\nu_{12}$ **	** $\nu_{13}$ **	** $\nu_{21}$ **
FEA	1.789	1.784	1.814	0.394	0.385	0.393
Two-step	1.786	1.764	1.797	0.394	0.386	0.391
Relative error	0.19%	1.11%	0.92%	0.08%	0.23%	0.53%
	** $\nu_{23}$ **	** $\nu_{31}$ **	** $\nu_{32}$ **	**$G_{23}$ [GPa]**	**$G_{13}$ [GPa]**	**$G_{12}$ [GPa]**
FEA	0.401	0.390	0.407	0.697	0.656	0.650
Two-step	0.403	0.387	0.414	0.708	0.657	0.652
Relative error	0.65%	0.91%	1.65%	1.52%	0.24%	0.28%
** $\theta =90^{\circ },\alpha =60^{\circ }$ **	**$E_{11}$ [GPa]**	**$E_{22}$ [GPa]**	**$E_{33}$ [GPa]**	** $\nu_{12}$ **	** $\nu_{13}$ **	** $\nu_{21}$ **
FEA	1.823	1.777	1.938	0.414	0.355	0.404
Two-step	1.832	1.776E	1.950	0.414	0.353	0.404
Relative error	0.46%	0.06%	0.62%	0.00%	0.43%	0.07%
	** $\nu_{23}$ **	** $\nu_{31}$ **	** $\nu_{32}$ **	**$G_{23}$ [GPa]**	**$G_{13}$ [GPa]**	**$G_{12}$ [GPa]**
FEA	0.385	0.377	0.419	0.694	0.635	0.633
Prediction	0.383	0.373	0.424	0.714	0.636	0.634
Relative error	0.40%	1.03%	1.18%	2.83%	0.12%	0.25%

**Table 5:** Accuracy comparison between the EML model and the three base learners on the training set and test set.

Model	ET		XGBoost		LGBM		EML	
	Train	Test	Train	Test	Train	Test	Train	Test
$R^{2}$	0.981	0.932	0.983	0.943	0.984	0.946	0.988	0.952
MSE	5.089 $\times 10^{15}$	2.360 $\times 10^{16}$	7.629 $\times 10^{15}$	2.050 $\times 10^{16}$	3.538 $\times 10^{15}$	1.450 $\times 10^{16}$	2.545 $\times 10^{15}$	1.260 $\times 10^{16}$
MAPE	0.831%	1.010%	0.716%	1.243%	0.971%	1.264%	0.567%	0.906%

**Table 6:** Accuracy evaluation of predictions by the EML model on a testing sample.

Metrics	$C_{11}$	$C_{12}$	$C_{13}$	$C_{14}$	$C_{15}$	$C_{16}$	$C_{22}$
$R^{2}$	0.99998	0.99999	0.99999	0.91273	0.95564	0.91864	0.99998
MSE	1.99 $\times 10^{16}$	1.36 $\times 10^{16}$	1.09 $\times 10^{16}$	8.8 $\times 10^{15}$	2.57 $\times 10^{15}$	9.94 $\times 10^{15}$	2.38 $\times 10^{16}$
MAPE	0.336%	0.533%	0.708%	0.671%	0.773%	1.063%	0.347%
	** $C_{23}$ **	** $C_{24}$ **	** $C_{25}$ **	$C_{26}$	$C_{33}$	$C_{34}$	** $C_{35}$ **
$R^{2}$	0.99999	0.92600	0.92918	0.94278	0.99996	0.94169	0.91682
MSE	1.04 $\times 10^{16}$	6.36 $\times 10^{15}$	7.65 $\times 10^{15}$	3.74 $\times 10^{15}$	5.64 $\times 10^{16}$	3.65 $\times 10^{15}$	1.30 $\times 10^{16}$
MAPE	0.587%	0.939%	0.809%	1.005%	0.812%	1.218%	1.010%
	** $C_{36}$ **	** $C_{44}$ **	** $C_{45}$ **	$C_{46}$	$C_{55}$	$C_{56}$	** $C_{66}$ **
$R^{2}$	0.91887	0.99929	0.90912	0.92698	0.99920	0.92795	0.99923
MSE	1.18 $\times 10^{16}$	4.46 $\times 10^{16}$	1.8 $\times 10^{15}$	1.5 $\times 10^{15}$	5.04 $\times 10^{15}$	2.31 $\times 10^{15}$	4.82 $\times 10^{15}$
MAPE	1.114%	0.917%	1.215%	1.981%	0.924%	1.179%	0.881%

**Table 7:** The microstructural parameters of SFRPCs of PA15 and PA6GF35.

Polymer Composite	$E_{m}\left[\mathrm{GPa}\right]$	$\nu_{m}$	$E_{f}\left[\mathrm{GPa}\right]$	$\nu_{f}$	d	a	VF	a_11_	a_22_	a_33_
PA15 from [46]	2.8	0.4	7.0	0.2	13.5	31.85	0.064	0.507	0.473	0.020
PA6GF35 from [47]	3.0	0.4	7.2	0.22	10	25	0.193	0.786	0.196	0.018

**Table 8:** Model efficiency comparison between the EML model and Digimat-FE calculations.

Method	Setting	Computation Cost
Digimat-FE Simulaiton	19,937 elements	2911 s
EML prediction	training 378 s (10,800 samples)	<1 s

**Figure A3:**
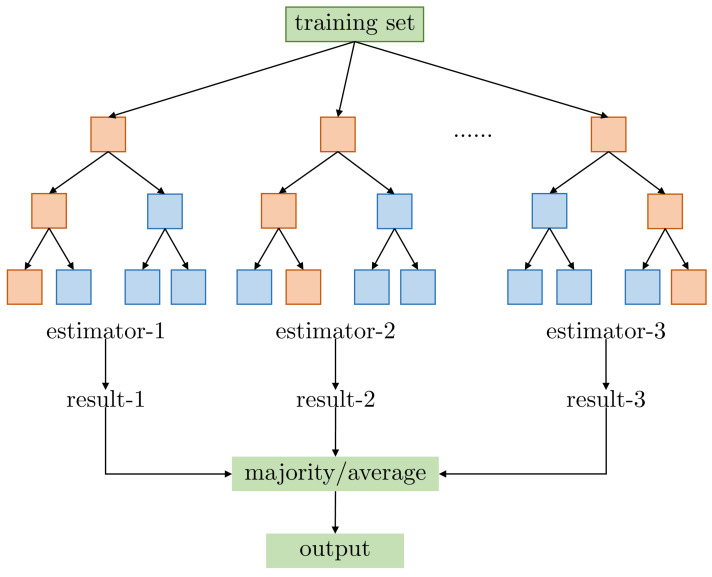
Shematic diagram of Extra Trees.

**Figure A4:**
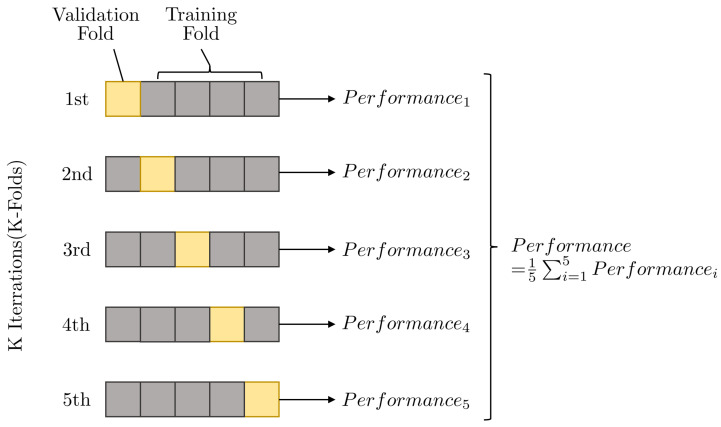
Schematic diagram for the K-fold cross-validation with K = 5.

## Data Availability

The data that support the findings of this study are available upon reasonable request from the corresponding authors.

## Appendix B. Preliminaries on ML Models Used in This Study

Three ML models are introduced according to their distinguished architecture, namely, ET, XGBoost, and LGBM.

### Appendix B.1. ET

ET [72] is a machine learning ensemble algorithm whose basic component is a decision tree.

For regression, the average of the outputs of all decision trees is used as the final output of ET. Every decision tree in the ensemble functions as an “individual learner” given it is an ensemble algorithm (see Figure 1 in Ref. [73]). The accuracy of ensemble learning is higher when the number of individual learners is fixed and their level of independence is higher.

As noted in Figure A3, the ET model is established according to the following strategy: (1) All the samples from the training dataset are used to create each decision tree, while the sample’s feature selection is done at random; (2) When splitting a node, for each non-constant feature contained in that node, an arbitrary number between the maximum and minimum value of the feature is randomly selected. (3) The sample is divided into the left branch if the feature value in it is greater than this value; otherwise, it is divided into the right branch. The samples in the node are then randomly assigned to the two branches covered by this feature. All the features in the node are traversed, and splitting values of all the features are obtained as described above. The node splits using the form with the highest splitting value. The ensemble learning model is improved as a result of the random processing approach used by ET to increase the independence of each decision tree.

For the detailed mathematical theory of ET, please refer to Ref. [72], which will not be introduced further in this paper.

### Appendix B.2. XGBoost

XGBoost is one of the most popular boosting tree algorithms for gradient boosting machines (GBM). Due to its outstanding problem-solving abilities and low demands on feature engineering, it has seen widespread application in the industrial sector. The fundamental idea of XGBoost is to continuously add and train new trees to fit residual errors from the previous iteration. The training error and the regularization, written as follows, constitute the objective function of XGBoost [64]:

(A1) Obj(θ)=∑L(θ)+∑Ω(θ)

where *L* represents the loss function used to calculate how far the projected values deviate from the actual values. Ω measures the training model’s complexity to prevent overfitting.

(A2) $$\Omega \left(\theta \right)=\gamma T+\frac{1}{2}\lambda {∥\omega ∥}^{2}$$

where *T* denotes the total number of leaf nodes and ω reflects the score of each leaf node. γ and λ are controlling factors used to prevent overfitting. overfitting.

When a new tree is created to fit the residual errors of the last iteration, the objective function is therefore approximated via an appropriate function:

(A3) $$L^{\left(t\right)}=\sum_{i=1}^{n}l\left(y_{i},{\hat{y}}_{i}^{\left(t-1\right)}+f_{t}\left(x_{i}\right)\right)+\Omega \left(f_{t}\right)$$

(A4) $$L^{\left(t\right)}\approx \sum_{i=1}^{n}\left[l\left(y_{i},{\hat{y}}_{i}^{\left(t-1\right)}\right)+g_{i}f_{t}\left(x_{i}\right)+\frac{1}{2}h_{i}f_{t}^{2}\left(x_{i}\right)\right]+\Omega \left(f_{t}\right)$$

where $g_{i}$ is the first-order derivative and $h_{i}$ denotes the second-order derivative.

Since each instance will eventually be assigned to a single leaf node, all instances that are members of that leaf node can be put back together as:

(A5) $${\mathrm{Obj}}^{\left(t\right)}\approx \sum_{j=1}^{T}\left[\left(\sum_{i\in I_{j}}g_{i}\right)w_{j}+\frac{1}{2}\left(\sum_{i\in I_{j}}h_{i}+\lambda \right)w_{j}^{2}\right]+\gamma$$

Therefore, the objective function can be derived as:

(A6) $$\mathrm{Obj}=-\frac{1}{2}\sum_{j=1}^{t}\frac{G_{j}^{2}}{H_{j}+\lambda }+\gamma T$$

### Appendix B.3. LGBM

LGBM itself is a boosting ensemble model that transforms coupled weak learners into a potential model, and such an algorithm has been provided as open-source since 2017 [74]. LGBM enhances the capabilities of Gradient-Boosted Decision Trees (GBDT) models in terms of increasing the efficiency of the model and reducing memory usage.

The model employs a histogram-based algorithm to mitigate the effects of high dimensional data, accelerate the computational time, and prevent the forecasting system from overfitting. This boosting technique consists of transforming the continuous floating-point eigenvalues into l integers and builds a histogram shape with depth restrictions and k width. It adopts a presorted-based Decision Trees (DT) technique. The training of LGBM additionally utilizes parallel learning using a parallel voting DT. For selecting the top-k samples, the initial samples are distributed into multiple trees with the Local Voting Decision (LVD) algorithm. The top-k LVD attributes are gathered by the global voting decision in order to calculate the top-k-2k attributes for the k iterations procedure.

The Leaf-wise approach is used by LGBM during the optimization phase to identify suitable leaves. The objective function of LGBM is given as [75]:

(A7) Obj(t)=L(t)+Ω(t)+c

(A8) $$L\left(t\right)=\sum_{n=1}^{n}{\left(y_{i}\left(t\right)-{\left(\hat{y}\right)}_{i}\left(t\right)\right)}^{2}$$

where Ω(t) and L(t) signify the regular and loss functions respectively, *c* stands for the extra parameter preventing overfitting and optimizing the depth of the tree, and *t* denotes the sampling time. The loss function L(t) represents the fitness of the model from the comparison of real value $y_{i}$, and predicted output ($\hat{y}{)}_{i}\left(t\right)$ for N samples.

## Appendix C. Hyperparameter Optimization

A hyperparameter is a characteristic of a model that is external to the model and whose value cannot be estimated from data. The value of the hyperparameter has to be set before the learning process begins.

### Appendix C.1. Grid search

Hyperparameters can be optimized using a variety of techniques, such as grid search, random search, bayesian optimization, etc. [76,77].

This work utilizes the grid search as the tuning technique to seek and determine the most suitable hyperparameter values. The method simply makes a complete search over a given subset of the hyperparameter space of the training algorithm (see Figure 4 in [77]). Given that the parameter space for some parameters in the machine-learning method may include spaces with real or unlimited values, it is exceedingly likely that users will need to set a boundary in order to use a grid search. Grid search suffers from high dimensional spaces, but often can easily be parallelized since the hyperparameter values that the algorithm works with are usually independent of each other.

Please see [36,77] for more information on the grid search method for machine learning models.

### Appendix C.2. K-Fold Cross-Validation

The goal of cross-validation is to estimate mate generalization error. The K-fold cross-validation method is used to assess the reliability of the machine learning model. As illustrated in Figure A4, the dataset for the study case is averaged and divided into K folds by using the stratified random sampling method. One of them is used as the validation set and other (K-1) folds are used as the training set. The process should be repeated K times, in order that each of the K folds is used as the validation set once. Then, the cross-validation error can be calculated and evaluated.

Mode details for cross-validation for machine learning models, please refer to References [36,77].

### Appendix C.3. Hyperparameters for the Base Learners

Each machine learning model typically has a multitude of hyperparameters, and most of these machine learning algorithms come with the default values of their hyperparameters. Adjusting other hyperparameters often yields minimal changes in the model’s predictive performance. Some of the default values perform well on the project, and only some key hyperparmeters truly impact the optimization process [36,78].

ET is an ensemble machine learning algorithm based on decision trees, and the main hyperparameters of ET include [72]:

- n_estimators is the number of decision trees.
- max_depth is the max depth of trees.
- min_samples_split is the least quantity of samples required for leaf splitting
- min_samples_leaf is the minimum number of sample numbers after child node splitting
- max_features is the number of features considered for a split.

XGBoost algorithm provides a large range of hyperparameters [64,79].

- n_estimators controls the number of trees in the model. Increasing this value generally improves model performance, but can also lead to overfitting.
- max_depth is used to limit the tree depth explicitly.
- learning_rate determines the step size at each iteration while moving toward a minimum of a loss function.
- gamma is the minimum loss reduction to create a new tree-split.
- min_child_weight is the minimum sum of instance weight needed in a child node.
- subsample denotes the proportion of random sampling for each tree.
- colsample_bytree is the subsample ratio of columns when constructing each tree.

The three main hyperparameters in XGBoost are the learning rate, the number of trees, and the maximum depth of each tree. These hyperparameters control the overall behavior and performance of the model.

LGBM uses the leaf-wise tree growth algorithm [79,80].

- num_leaves is the maximum number of leaves, the main parameter to control the complexity of the tree model.
- min_data_in_leaf very important parameter to prevent over-fitting in a leaf-wise tree.
- max_depth is used to limit the tree depth explicitly.
- n_estimators is the number of boosted trees to fit.
- learning_rate controls the step size at which the algorithm makes updates to the model weights.
- colsample_bytree is the subsample ratio of columns when constructing each tree.
- subsample dictates the proportion of random sampling for each tree.

For more introductive information on hyperparameters of machine-learning models, please refer to References [36,60,76,81].
